# Interfractional anatomic variation in patients treated with respiration‐gated radiotherapy

**DOI:** 10.1120/jacmp.v6i2.2048

**Published:** 2005-05-21

**Authors:** Ellen Yorke, Kenneth E. Rosenzweig, Raquel Wagman, Gikas S. Mageras

**Affiliations:** ^1^ Department of Medical Physics Memorial Sloan Kettering Cancer Center 1275 York Avenue New York City New York 10021 U.S.A.; ^2^ Department of Radiation Oncology Memorial Sloan Kettering Cancer Center 1275 York Avenue New York City New York 10021 U.S.A.

**Keywords:** gating, respiration, radiotherapy

## Abstract

As quality assurance for respiration‐gated treatments using the Varian RPM™ system, we monitor interfractional diaphragm variation throughout treatment using extra anterior‐posterior (AP) portal images. We measure the superior‐inferior (SI) distance between one or more bony landmarks and the ipsilateral diaphragm dome in each such radiograph and calculate its difference, *D*, from the corresponding distance in a planning CT scan digitally reconstructed radiograph (DRR). For each patient, the mean of *D* represents the systematic diaphragm displacement, and the standard deviation of *D* represents random diaphragm variations and is a measure of interfractional gating reproducibility. We present results for 31 sequential patients (21 lung, 10 liver tumors), each with at least 8 such portal images. For all patients, the gate included end‐exhale. The patient‐specific duty cycle ranged from 30% to 60%. All patients received customized audio prompting for simulation and treatment, and 14 patients also received visual prompting. Respiration‐synchronized fluoroscopic movies taken at a conventional simulator revealed patient‐specific diaphragm excursions from 1.0 cm to 5.0 cm and diaphragm excursion within the gate from 0.5 cm to 1.0 cm, demonstrating a significant reduction of intra‐fractional diaphragm (and by inference tumor) motion by respiratory gating. One standard deviation of the systematic displacement (the mean of *D*) was 0.63 cm and 0.48 cm for the lung and liver patient groups, respectively. The average ±1 SD of the random displacements (i.e., the average of the standard deviations of *D*) was 0.42±0.11 cm and 0.50±0.19 for the two groups, respectively. The similar magnitude of the systematic and random displacements suggests that both derive from a common distribution of interfractional variations. Combining visual with audio prompting did not significantly improve performance, as judged by *D*. Guided by these portal images, field changes were made during the course of treatment for 6 patients (1 lung, 5 liver).

PACS numbers: 87.53.‐j, 87.53.Oq

## I. INTRODUCTION

Respiratory motion of thoracic and abdominal tumors can exceed 2 cm, which compromises the accuracy of three‐dimensional conformal radiation therapy at these sites. For current surveys of thoracic tumor motion studies, see Langen and Jones,^(1)^ Mechalakos et al.,^(2)^ and references therein. To prevent underdosing of a target that undergoes respiratory motion during simulation and treatment, an extra safety margin is included in the planning target volume (PTV). However, this includes excess normal tissue, which causes increased risk to normal tissues and/or a reduction in prescription dose.

Respiratory gating is a technique for limiting the adverse effects of this motion by acquiring planning images and delivering therapy beams only during a selected portion of the breathing cycle (the gate), which is determined by the signal from a breathing monitor. Ideally, when the monitor signal is within the gate, the tumor and normal anatomy are in the same position (within user‐specified tolerances) at simulation and for all treatments. A variety of breathing monitors have been suggested and used.^(3)^–^(9)^ One such is the Varian RPM™ system (Real‐time Position Management, Varian Medical Systems, Palo Alto, CA), which has been the subject of several reports.^(10)^–^(17)^ The RPM system monitors respiratory motion using the motion of markers on the patient's surface. A key assumption underlying this method is that reproducible marker motion implies reproducible tumor motion. Ideally, this would be verified by direct observation of the tumor, but if the tumor is poorly visualized on portal images, the diaphragm is often used as a clearly visible surrogate. The motion of liver tumors in the superior‐inferior (SI) direction is reported to correlate well with diaphragm motion.^(18)^ Good correlation between diaphragm and lung tumor motion has also been observed in some patients,^(19)^ but the more complicated motion of lung tumors makes further, patient‐specific study desirable.^(20)^

In fluoroscopic studies, we observed good correlation of marker and diaphragm motion and good intra‐fractional reproducibility of the SI location of the apex of the diaphragm^(11)^ with RPM gating. However, intra‐fractional reproducibility does not necessarily imply reproducibility over 4 to 8 weeks of treatment, since respiratory patterns and internal anatomy can change due to co‐morbid conditions, therapy response, disease progression, or breathing changes as the patient adapts to the treatment routine. For the first 8 patients treated with RPM gating at our institution, the mean displacement of the diaphragm on localization films taken at treatment differed from the value at simulation by more than 0.4 cm for 4 patients.^(12)^ Subsequently, as quality assurance of all RPM‐gated treatments in our department, we acquire gated anterior‐posterior (AP) portal images 1 to 3 times per week throughout the course of treatment and measure the SI distance between isocenter and the dome of the ipsilateral diaphragm, corrected for SI setup error. Below, we report on the interfractional variations in a larger and later group of 31 sequential patients treated with RPM gating.

## II. METHODS

### A. RPM system

In the RPM gating system, a pair of infrared reflective markers rigidly mounted in a plastic block is placed on the patient's chest. The motion of these markers is monitored by a CCD camera with an attached infrared illuminator. The camera output goes to a PC running the RPM software. After a “tracking” algorithm establishes the period and amplitude of the marker motion, the motion is “recorded” and displayed as a graph (the marker motion trace) on the PC monitor. In this study, we used amplitude gating, where the user sets amplitude thresholds that define the range of marker positions within the gate. At a conventional simulator, fluoroscopic movies can be taken in synchrony with the marker motion, saved, and played back to observe anatomical motion within any chosen gate. At a CT‐simulator, a signal from the RPM system triggers slice acquisition in axial mode when the motion enters the gate. For treatment, a signal from the PC to the gun grid enables the beam only when the motion is within the gate.

**Figure 1:**
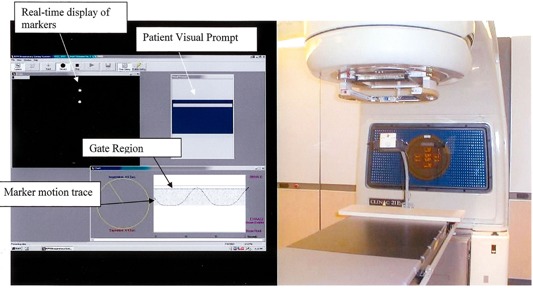
The RPM screen and visual feedback hardware. (a) The RPM v1.5 PC screen for amplitude‐gated treatment or simulation at end‐inhale. The treatment beam would be on when the marker motion trace is between the two horizontal lines labeled “Gate Region.” (b) The small LCD monitor mounted on the couch, on which the patient can view the visual prompt

The RPM data from the planning CT is the reference session for treatment. To encourage regular breathing, customized voice instruction is produced by adjusting pause lengths in a recorded phrase, “breathe in (pause1), breathe out (pause2),” to a rhythm that is comfortable for the patient. To implement visual prompting, which has been suggested to further improve breathing regularity,^(17)^,^(19)^ an amplitude typical of the marker excursion at simulation is chosen. This is displayed as a cross‐hatched region in the marker motion trace and as a solid rectangle in a simpler display for the patient. This amplitude and the audio instructions are saved in the reference session and provide breathing constancy cues for the patient and the therapists at treatment. In our clinic, the patient views the visual prompt on a couch‐mounted monitor ((Fig. 1b) and is trained to make the bar touch the top of the blue rectangle at end‐inhale and the bottom at end‐exhale. The therapist sees the full display on the PC and can supervise breathing regularity. A typical PC screen is shown in (Fig. 1a).

## B. Simulation and treatment planning

We analyzed the portal images for 31 sequential patients treated with RPM respiratory gating. This study has been approved by our Institutional Review Board. There were 21 nonsmall cell lung cancer patients, 9 patients with liver cancer (primary or metastases) and one with a rhab‐domyosarcoma near the liver. All were outpatients, treated with curative or long‐term palliative intent. The average lung patient age was 67.6 years (range 54 to 83 years), with 11 males and 10 females. The average liver patient age was 55 years (range 17 to 80 years), with 6 males and 4 females. A graticule tray projecting a 2‐cm square grid at isocenter was used for all films, and a similar grid was overlaid on the digitally reconstructed radiographs (DRRs) to facilitate absolute distance measurements and locate isocenter.

Before simulation, each patient was immobilized supine, with arms above the head, in a custom foam cradle (Alpha Cradle Molds, Akron, OH). The marker block was between umbilicus and xiphoid, at a location where its motion amplitude was at least 0.5 cm. All patients were trained to follow the customized voice instruction described above, and 16 were trained and simulated with the addition of visual prompting. Because patients breath more deeply when following voice instruction,^(11)^ they were encouraged to “breath normally” and to not hyperventilate. The same marker block location and breathing instructions were used for CT simulation and treatment. At an RPM‐equipped simulator (Ximatron, Varian Medical Systems, Palo Alto, CA), anterior fluoroscopic movies of the ipsilateral diaphragm (estimated skin exposure 2.5 R for a 45‐s movie) were acquired for 27 of the patients. If the tumor was visible, its motion amplitude was also noted. For all patients, the gate interval for acquisition of the planning CT images and treatment included end‐exhale. The gate width was based on the fluoroscopic movies and the motion trace so that the duty cycle (percent of breathing cycle within the gate) was at least 25%. Gated AP simulator films or computed radiographs (Kodak RT3000, Rochester, NY) were acquired at the isocenter set by the radiation oncologist.

Planning CT images were acquired on an RPM‐equipped PQ 5000 (Philips Marconi Medical Systems, Cleveland, OH). Slice separation and thickness were 5 mm for 28 patients and decreased to 3 mm for the most recent 3 patients. Patients who participated in an imaging study also received a respiration‐triggered scan at end‐inspiration^(21)^ or a respiration‐correlated CT scan.^(22)^–^(25)^ For all patients, high‐contrast DRRs of the treatment fields and an orthogonal pair were generated as well as a “soft tissue” AP and/or PA DRR (same mass attenuation coefficient for all tissues). These resemble megavoltage portal images and are reference images for the diaphragm position during gated treatment. The treatment fields were selected by the planner and did not have to include AP or PA beams. A volumetric, patient‐specific CTV to PTV margin of 1 cm to 1.5 cm for lung patients^(26)^ and 1 cm for the liver patients^(21)^ was used for planning, but this could be increased partway through treatment depending on observed interfraction variability.

### C. Diaphragm radiographs

All patients were treated with RPM gating on a Varian 2100‐EX LINAC. The therapists gave extra instruction to patients who had difficulty with regular breathing and reported persistent problems to a physician or physicist. All portal imaging was gated and used 6‐MV photons. In addition to the weekly treatment field portal images, extra open AP or PA radiographs showing the ipsilateral diaphragm, isocenter, and spine (hereafter called “diaphragm films”) were acquired. Four monitor units (MU) were used per extra film to restrict the imaging dose (our double‐exposure treatment field films are 6 to 8 MU). For the first two weeks of treatment, we requested diaphragm films for at least 5 sessions. If no problems were encountered, the frequency of these films was reduced to twice per week in weeks 3 and 4 and weekly thereafter. Most diaphragm imaging used film or computed radiography because electronic portal imaging with RPM versions 1.3 to 1.5 requires physics assistance. However, electronic imaging was done for lung patients LU‐13 (5 days), LU‐14 (1 day), and LU‐15 (3 days). Each electronic portal imaging device (EPID) session used a total of 4 MU but provided 3 to 5 diaphragm images.

For each diaphragm film, we visually chose as an origin a prominent vertebral feature that was also visible on the DRR or the gated AP simulator film. It was sometimes necessary to use different features for different diaphragm films because a feature visible on one diaphragm film was obscured on another. The most prominent bony landmarks are also different from patient to patient. The only requirement for a reference feature was that it not move with respiration and be clearly identifiable on both the reference image and the diaphragm film. The DRRs and diaphragm films were digitized into an in‐house image review program. Measurements were done with the aid of an electronic ruler and the graticule. We determined the shift required to make the SI distance from isocenter to this feature the same as in the reference DRR and/or simulator film. We defined $d_{p}$ as the SI distance between the ipsilateral diaphragm apex and the shifted isocenter and calculated the difference between $d_{p}$ and the isocenter‐diaphragm distance, $d_{\text{ref}}$, in the reference image. We defined *D* as

(1a) $$D=d_{p}-d_{ref}\;for\;lung\;cancer\;patients\;and$$

(1b) $$D=d_{ref}-d_{p}\;for\;liver\;cancer\;patients$$

**Figure 2:**
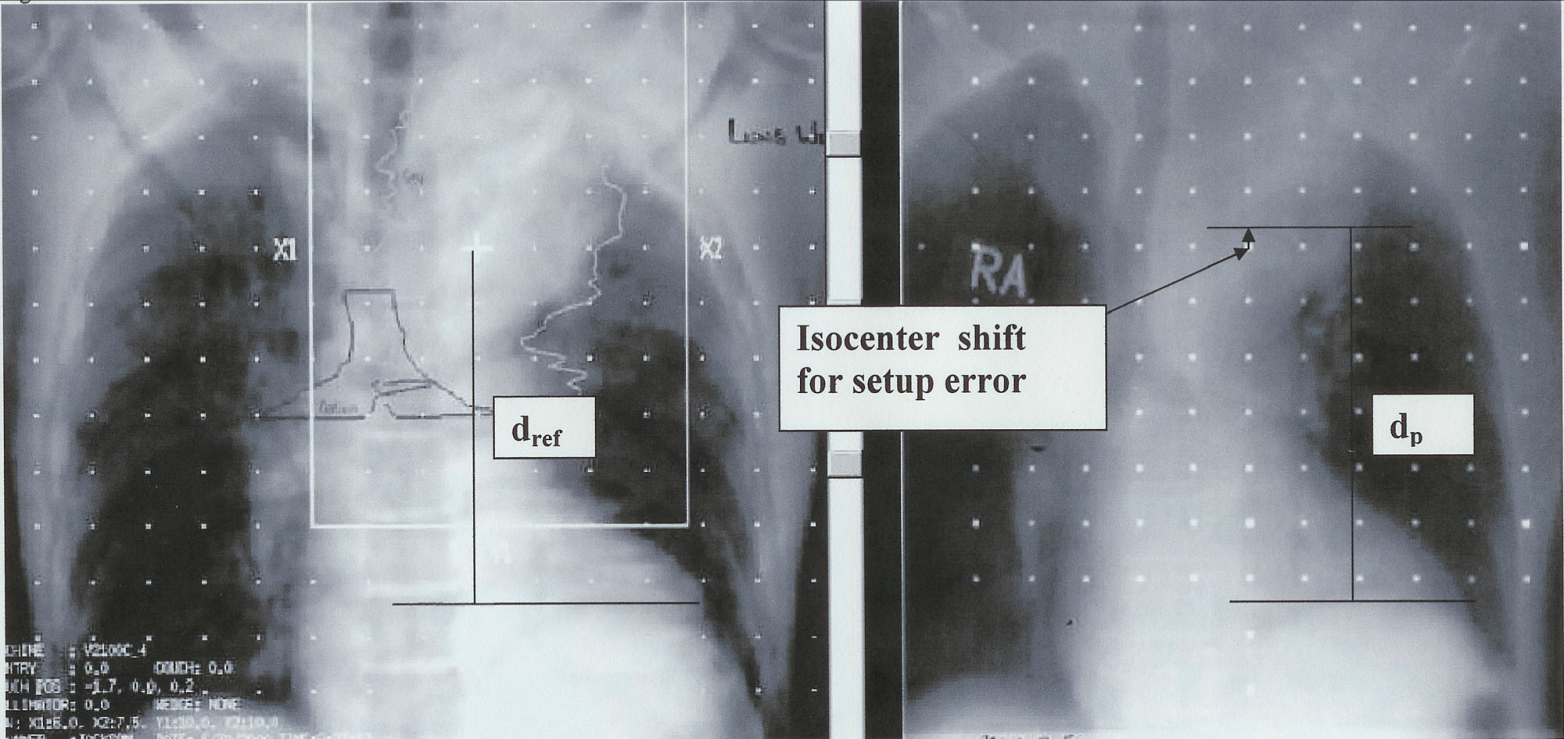
The determination of *D*. *D* is found by comparing the distance of isocenter to diaphragm on an AP DRR from the planning scan with the distance of isocenter to diaphragm on an AP or PA portal image. Correction for setup error on the portal image is made by finding the distance from isocenter to a vertebral landmark visible on both images.

*D* is positive if the diaphragm is inferior relative to the DRR for both the lung patients (isocenter superior to diaphragm) and the liver patients (isocenter inferior to diaphragm); *D* is negative for a superior diaphragm displacement. Ideally, *D* is not affected by setup error and depends only on the interfractional performance of gating. However, because small vertebral features are blurred by partial volume effects in the DRR and are often indistinct on thoracic port films, we did not attempt to correct setup errors less than 3 mm. Figure 2 illustrates the determination of *D*.

Both *D* and the “raw” isocenter‐diaphragm distance supply information on the adequacy of the PTV. If a large systematic or film‐to‐film variation of *D* was seen in the first 2 to 3 weeks, the case was discussed and sometimes replanned with a larger PTV. At the end of treatment, $D_{\text{pt}}$, the mean value of *D*, and $S_{\text{pt}}$, the standard deviation of *D*, were calculated for each patient. $D_{\text{pt}}$ represents the systematic change in diaphragm position compared to simulation. $S_{\text{pt}}$ is determined by random positional variation of the diaphragm and is a measure of interfraction gating reproducibility.

## III. RESULTS

All patients had between 8 and 16 diaphragm films. Approximately 20% of these had SI setup error exceeding 3 mm. The average value of *D* over all the patients was −0.12 cm with the standard error in the mean of ±0.1 cm. The average of the standard deviations ($S_{\text{pt}}$'s) over all patients was 0.45 cm with standard error in the mean of ±0.03 cm. Breathing periods ranged from 4 s to 6 s, and duty cycles ranged from 27% to 55%; breathing traces of patients with large duty cycles were characterized by a long, flat exhale plateau. Tables 1 and 2 summarize the data for the individual lung and liver patients, respectively.

**Table 1:** Summary for lung cancer patients. Positive $D_{\text{pt}}$ means portal image diaphragm is inferior to DRR (more like “inhale” than simulation). $S_{\text{pt}}$ is the SD of D for an individual patient. The estimated error in $D_{\text{pt}}$ is $S_{\text{pt}}/\sqrt{\left(\#\;\text{films}\right)}$. Diaphragm excursions are estimated visually from a fluoroscopic movie acquired at simulation. The approximate period and duty cycle are estimated from the CT simulation reference session.

Patient	#film	Visual prompt	$D_{\text{pt}}\pm \text{estimated error}\;(\text{cm})$	$S_{\text{pt}}\;(\text{cm})$	Range of D (cm)	% within 0.5 cm of $D_{\text{pt}}$	Estimated diaphragm excursion (cm)	Estimated diaphragm excursion in gate (cm)	Period(s)	Duty cycle (%)
1_LU	9	no	0.14±0.09	0.27	(−0.2, 0.6)	100%	~1.5	not available^a^	5	38
2_LU	12	no	−0.05±0.11	0.38	(−0.6, 0.4)	92%	~1.5 to 2.0	~0.5	5	29
3_LU	11	no	−0.03±0.10	0.30	(−0.6, 0.4)	91%	~1.0 to 1.5	~0.5	4	40
4_LU	9	no	0.04±0.16	0.48	(−0.4, 1.2)	89%	~2.0	~0.5	4.5	27
5_LU	12	no	−1.14±0.14	0.48	(−1.6, 0.2)	92%	~2.5 to 3.0	~0.5	4	31
6_LU	12	yes	0.02±0.14	0.50	(−1.0,0 .9)	67%	~1.5 to 2.0	~0.7	5	50
7_LU	12	yes	−0.38±0.10	0.34	(−10, 0.1)	92%	~2.0	~0.5	5	35
8_LU	12	no	−1.12±0.09	0.32	(−1.6, −0.7)	100%	~2.0	~0.5	5	39
9_LU	16	yes	0.35±0.10	0.40	(−0.3, 1.1)	81%	~1.0–1.5	~0.5	5	36
10_LU	14	no	0.34±0.16	0.58	(−0.4, 1.6)	50%	no movie	no movie	5	47
11_LU	10	no	−0.13±0.13	0.40	(−1.0, 0.4)	80%	~4.0–5.0	not available	4	42
12_LU	9	no	−0.03±0.06	0.17	(−0.4, 0.2)	100%	no movie	no movie	5	46
13_LU	17	yes	1.04±0.14	0.56	(−0.01, 1.8)	65%	2.2	~0.8	4.4	38
14_LU	9	yes	−1.41±0.17	0.51	(−2.1, −0.3)	78%	1.5	~0.8	5	35
15_LU	12	yes	0.35±0.11	0.37	(−0.2, 0.8)	83%	3.3	~1.0	6	39
16_LU	11	yes	0.19±0.09	0.30	(−0.25, 0.55)	100%	2.3	~0.5	5.8	44
17_LU	10	no^b^	−1.37±0.17	0.54	(−1.75, −0.15)	90%	2.5	~0.7	5.2	42
18_LU	11	no^b^	0.05±0.17	0.57	(−0.7, 1.6)	82%	1.5	atypical trace^a^	5	55
19_LU	14	yes	0.19±0.14	0.51	(−0.5, 1.2)	71%	2.2	~1.0	4.7	51
20_LU	8	yes	−0.25±0.14	0.4	(−0.9, 0.4)	75%	1.5	~0.5	4.9	32
21_LU	11	yes	0.38±0.14	0.49	(−0.35, 1.25)	73%	1.7	~0.7 (atypical trace^a^)	5.4	54

^a^ Fluoro movie connection to breathing trace corrupted; breathing trace for fluoro qualitatively different from CT and treatment.

^b^ Offered visual prompting but declined

**Table 2:** Summary for liver cancer patients. Positive $D_{\text{pt}}$ means portal image diaphragm is inferior to DRR (more like “inhale” than simulation). $S_{\text{pt}}$ is the SD of D for an individual patient. The estimated error in $D_{\text{pt}}$ is $S_{\text{pt}}/\sqrt{\left(\#\;\text{films}\right)}$. For patient 3_Li, the tumor crossed midline, and both diaphragms were monitored (sometimes on different port films). The approximate period and duty are estimated from the CT simulation reference session

Patient	#film	Visual prompt	$D_{\text{pt}}\pm \text{estimated error in mean}\;(\text{cm})$	$S_{\text{pt}}\;(\text{cm})$	Range of D (cm)	% films with D within 0.5 cm of $D_{\text{pt}}$	Estimated diaphragm excursion (cm)	Estimated diaphragm excursion in gate (cm)	Period (s)	Duty cycle (%)
1_Li	14	no	0.29±0.13	0.49	(−0.7, 1.0)	64%	no movie	not available	not available	not available
2_Li	9	no	0.78±0.17	0.50	(0.2, 1.7)	67%	~3.0 to 4.0	~1.0	5.5	38
3_Li_R	9	no	−0.10±0.17	0.50	(−0.9, 0.7)	67%	~2.0 to 3.0	~0.9	5.4	34
3_Li_L	9	no	−0.20±0.22	0.67	(−1.6, 0.6)	67%	no movie	not available	(same pt)	
4_Li	11	no	−0.23±0.25	0.84	(−1.8, 1.0)	45%	~3.0 to 4 0	~1.0	4.5	35
5_Li	8	no	−0.01±0.12	0.35	(−0.5, 0.5)	88%	~2.5 to 3.0	~0.7	6	56
6_Li	8	yes	−0.49±0.18	0.50	(−1.4,0.0)	88%	~2.0 to 2.5	~0.7	5	32
7_Li	12	yes	−0.79±0.04	0.14	(−1.0, −0.5)	100%	no movie	not available	5	42
8_Li	15	no	−0.73±0.17	0.66	(−2.0, 0.3)	67%	~2.0	~0.6	6	30
9_Li	12	yes	0.20±0.09	0.32	(−0.3, 0.9)	92%	~1.0 to 1.5	~0.4	5	40
10_Li	14	yes	0.42±0.14	0.53	(0.2, 1.9)	79%	2	~04	6	55

### A. Lung cancer patients

Fluoroscopic movies were available for 19 of the 21 lung cancer patients, and breathing‐synchronized movies could be reconstructed for 17 lung patients. For 2 patients, the motion trace recorded along with the movie was atypical in that it differed greatly from the trace at CT and treatment. Without gating, the end‐exhale to end‐inhale ipsilateral diaphragm excursion estimated from the movies ranged from 1 cm to 5 cm, and gating reduced it to a mean of 0.6 cm (0.4 cm to 1 cm). These differences were statistically significant ($p<10^{-3}$, two‐sided *t*‐test).

**Figure 3:**
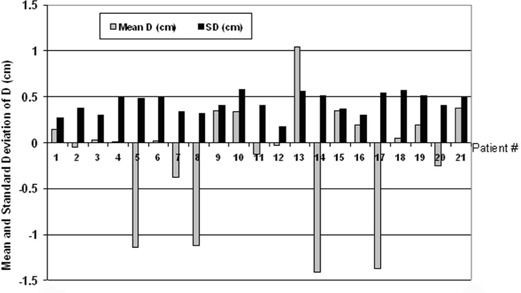
The mean and standard deviation of *D* ($D_{\text{pt}}$ and $S_{\text{pt}}$) for the lung cancer patients

**Figure 4:**
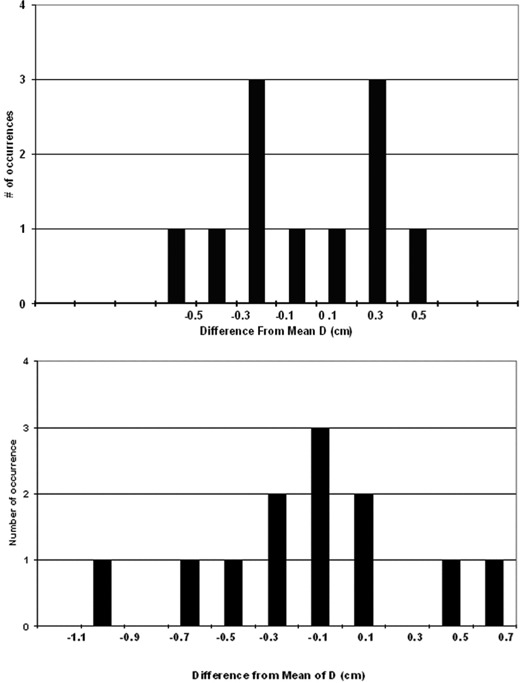
Frequency distribution of *D* for (a) patient 3_LU and (b) patient 6_LU

For each lung patient, the systematic deviation of the ipsilateral diaphragm position relative to simulation ($D_{\text{pt}}$) and the random film‐to‐film variation $\left(S_{\text{pt}}\right)$ are listed in Table 1 and plotted in Fig. 3. Averaging over the lung patients, the average±1 SD of $D_{\text{pt}}$ was−0.13±0.63 cm (range 1.41 cm superior to 1.04 cm inferior), and the average±1 SD of $S_{\text{pt}}$ was 0.42±0.11 cm (range 0.17 to 0.58 cm). For 16 patients, $D_{\text{pt}}$ was 0.5 cm or less, indicating that their average diaphragm position was within 0.5 cm of simulation. The other 5 patients (5_LU, 8_LU, 13_LU, 14_LU, and 17_LU) had systematic deviations from simulation exceeding 1 cm, although, as judged by the PC display, they were regular breathers with no notable change in marker motion relative to simulation. Patients 13_LU and 14_LU also had visual prompting throughout treatment. Patient 17_LU (also 18_LU) found visual prompting confusing, and it was discontinued early in treatment. Although the gate was centered approximately at end‐exhale, for 4 patients (5_LU, 8_LU, 14_LU, and 17_LU) the systematic diaphragm shift was superior $\left(D_{\text{pt}}<0\right)$ by more than 1 cm, suggesting a more pronounced exhale at treatment than at simulation. Five patients (2_LU, 4_LU, 6_LU, 11_LU, and 19_LU) had small systematic difference from simulation $\left(|D_{\text{pt}}|⩽0.2\;\text{cm}\right)$, but three of these (4_LU, 6_LU, and 19_LU) had larger than average film‐to‐film variations. For an individual patient, the distribution of *D*s did not visually appear to be Gaussian, as shown in (Figs. 4a)and (4b). Thus, as a second measure of diaphragm variability, Table 1 also lists the percent of each patient's diaphragm films with *D* within 0.5 cm of $D_{\text{pt}}$. This was all films for 4 patients and all but one film for 11 patients. Patient 5_LU was noted to have a large $S_{\text{pt}}$ (0.48 cm) but had *D* within 0.5 cm of $D_{\text{pt}}$ for 11 of the 12 films; $S_{\text{pt}}$ was dominated by a single film. This patient also had a large systematic difference from simulation, but the diaphragm position was consistent with one exception.

Repeat EPID images for patients 13_LU and 14_LU demonstrated the good intra‐fraction variability that has been reported previously,^(12)^ with a maximum intra‐fraction diaphragm variation of 0.3 cm for 13_LU and 0.5 cm for 14_LU. Patient 15_LU had three EPID imaging sessions. For two sessions, the maximum intra‐session variation was 0.3 cm. In the third session, 4 of 5 images had diaphragm positions within 0.5 cm of each other, but the largest difference in this session was 0.9 cm due to one outlier.

The PTV and beam apertures for 5_LU were increased. To do this, we assumed that the gross tumor volume (GTV) would shift rigidly in the SI direction by the same amount as the diaphragm and enlarged the PTV to cover both the planning and the shifted GTVs. The apertures were enlarged to cover the new PTV in beam's‐eye view. We enlarged apertures rather than shift the isocenter with fixed apertures because we could not predict diaphragm displacements later in the course of treatment, and we did not want to make repeated field changes. Because estimated normal tissue toxicity (lung and cord) was small for this patient, we did not change the prescription dose. Patients 8_LU and 13_LU had tumors that were visible on the portal images and were seen to be well within the field so no field changes were made. In addition, no field changes were made for 14_LU and 17_LU.

### B. Liver cancer patients

There were fluoroscopic movies for 8 of the 10 liver patients. Their free‐breathing diaphragm excursion ranged from 1 cm to 4 cm, and gating reduced it to an average of 0.7 cm (range 0.4 cm to 1 cm), a statistically significant reduction ($p<10^{-3}$). The difference in diaphragm excursion between the lung cancer and liver cancer patients was not statistically significant.

$D_{\text{pt}}$ ranged from 0.79 cm superior to 0.78 cm inferior of simulation. Averaging over the liver patients, D±1 SD was −0.08 cm±0.48 cm, and the average ±1 SD of $S_{\text{pt}}$ was 0.50±0.19 cm. Although the gate was around end‐exhale, 3 patients (6_Li, 7_Li, and 8_Li) had systematic diaphragm shifts superior of simulation. For 7 patients, $D_{\text{pt}}$ was within 0.5 cm of simulation, but 3 of these had large film‐to‐film variation as indicated by $S_{\text{pt}}$ and the fact that more than 30% of their films had *D* over 0.5 cm different from $D_{\text{pt}}$. $S_{\text{pt}}$ ranged from 0.14 cm to 0.84 cm, and for only one patient (7_Li) was *D* within 0.5 cm of $D_{\text{pt}}$ for all the films. Although this patient had a large systematic deviation from simulation, the value of *D* was consistent over the course of treatment. The difference between $D_{\text{pt}}$ and $S_{\text{pt}}$ for the liver and lung patients is not statistically significant.

**Figure 5:**
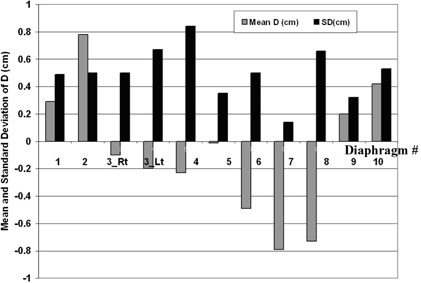
The mean and standard deviation of *D* ($D_{\text{pt}}$ and $S_{\text{pt}}$) for the liver cancer patients. Note that there are two sets of bars for patient 3, for whom both right and left diaphragms were monitored.

The liver cancer patients' results are tabulated in Table 2, and $D_{\text{pt}}$ and $S_{\text{pt}}$ are plotted in Fig. 5. The target volume of patient 3_Li crossed midline, so the displacements of both diaphragms are included. Field and treatment plan changes based on the diaphragm films were made during treatment for five patients: 2_Li, 3_Li, 4_Li, 7_Li, and 8_Li. As described for patient 5_LU, we created new plans with enlarged PTVs and apertures. However, except for patient 7_Li, we also decreased the total prescription dose to respect liver tolerance.

### C. Visual prompting

The average±1 SD of $D_{\text{pt}}$ over the 14 patients who had both audio and visual prompting throughout treatment was−0.02±0.6 cm, and over those with audio prompting only, it was0.19±0.56 cm. The two patients who declined visual prompting are included in the audio‐only group. The average±1 SD of $S_{\text{pt}}$ over the patients with both types of prompting was 0.42±0.12 cm and over those with audio prompting only, was 0.47±0.16 cm. These differences were not statistically significant (two‐sided *t*‐test).

### D. Clinical feasibility

The time to acquire and evaluate diaphragm films is not excessive, amounting to less than 5 min per film if only isocenter‐to‐diaphragm distance is examined. A film adds less than a minute to the patient's in‐room time. The raw change in isocenter‐to‐diaphragm distance relative to simulation (no correction for setup error) can be rapidly determined by visual inspection aided by the grid. Determining setup error relative to bony landmarks can be time‐consuming, depending on port film and DRR quality, but radiation oncologists can efficiently judge whether the setup error is clinically acceptable. In this study, setup error was examined with particular care in order to separate the interfractional performance of the RPM system from setup error. For purely clinical purposes, the raw isocenter‐diaphragm distance gives sufficient information providing that setup error is within the physician's tolerance. The most time‐consuming part of the process is discussing and replanning patients. However, we feel this is necessary to evaluate and, if possible, correct systematic errors introduced by respiratory gating based on an external marker. Extra time required for gated simulation and treatment^(14)^,^(17)^ is an accepted cost of respiratory gating and is beyond the scope of this study of interfractional changes.

## IV. DISCUSSION

Previous studies of respiratory gating with the RPM system demonstrated good intra‐fraction reproducibility of the positions of diaphragm^(12)^ and other thoracic organs^(21)^ as indicated by repeat images during a single session. However, the goal of respiratory gating is to reproduce tumor and normal tissue positions from simulation throughout the course of treatment. Our observations were qualitatively similar to those of Ford et al.^(12)^ for the first 8 RPM‐gated patients (4 liver, 4 lung) at our institution. They found that the absolute value of $D_{\text{pt}}$ exceeded 0.5 cm for 1 patient and exceeded 0.4 cm for 4, and that the average of $S_{\text{pt}}$ over the 8 patients was 0.28 cm. For one patient with gate centered around end‐exhale, $D_{\text{pt}}$ was systematically superior of simulation by 0.6 cm. Because these results were obtained early in our clinical gating experience, they might have been adversely affected by inexperience or positively affected by strict physics oversight. For the patients in the current study, gating was integrated into the clinical process, and diaphragm filming continued for the entire course of treatment. In this study, we found that the absolute value of $D_{\text{pt}}$ exceeded 0.4 cm for 10 of 31 patients, and the mean diaphragm position was systematically superior of its simulation position by 0.5 cm or more for 6 patients.

**Figure 6:**
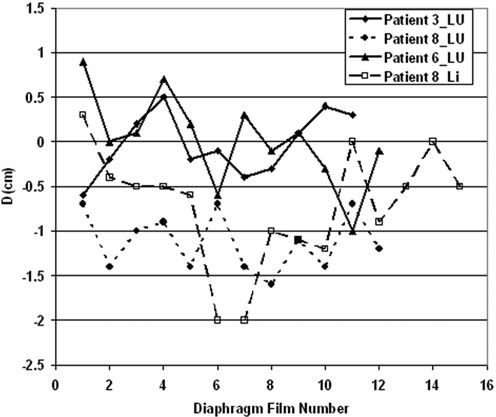
Chronological variation of *D* over the course of treatment for four patients. These show four qualitatively different types of variation in diaphragm position.

We observed a variety of patient‐specific patterns of diaphragm variability, as shown in Fig. 6, a chronological plot of *D* over the course of treatment for 4 patients. The preferred behavior, shown by patient 3_LU, is a small systematic deviation and a small interfraction variation. However, some patients have a large systematic deviation from simulation with a small interfraction variation, as shown by patient 8_LU. There are also cases (patient 6_LU) with a large interfraction variability where superior and inferior displacements approximately cancel, resulting in a small systematic displacement. The most difficult cases (patient 8_Li) have both large interfraction variability and large systematic displacement.

Because there were almost four times more patients in this study than in the study reported in Ref. 12, we could more confidently examine the population distribution of *D*. The systematic and random deviations of *D* for the lung and liver patients are of similar magnitude. One interpretation of this observation is that there is a distribution of possible diaphragm positions that governs both simulation and treatment. Simulation is a single sample from the distribution, and each treatment is another sample. This distribution need not be normal. The four behaviors shown in Fig. 6 would follow from such a model.

Our expectation that the diaphragm films and the DRRs image approximately the same part of the breathing cycle for breathing periods (4 s to 6 s) and duty cycles (27% to 55%) characteristic of our patients is based on two estimates. First, the average of *D* over the entire patient set is close to zero (−0.12 cm), indicating that little or no systematic bias is introduced by comparing diaphragm positions on the DRRs and the diaphragm films. Second, the diaphragm spends similar time in the gate for the CT and portal images. Slice acquisition on the PQ500 starts 330 ms after the breathing trace enters the gate and takes 1 s. For a 5‐s breathing period and a 33% duty cycle, 1.7 s is spent in the gate, and the slice is acquired approximately equally about the center of the gate interval. For port films, the beam turns on when the trace enters the gate. At the port‐film mode dose rate of 100 MU/min, a 4 MU diaphragm film takes about 2.4 s (1.4 gate intervals), so the film certainly includes the end‐exhale position of the diaphragm apex. For both film and DRRs, the apex density depends on how quickly it moves through end‐exhale, an effect that may bias both film and DRR toward an estimated position inferior to true end‐exhale.

We used customized voice instruction for all patients for two main reasons, as described in Ref. 11. First, it substantially reduces the fraction of treatment time that the beam is held off due to irregular breathing. Second, it reduces cycle‐to‐cycle variation in the marker position at end expiration, thus reducing the likelihood of waveform drift with respect to the gating thresholds. Thus, it helps the patient breath regularly throughout the course of treatment. Although total breathing amplitude is increased, in Ref. 11 we found that the diaphragm excursion within the gate is not adversely affected by the larger amplitude but rather is comparable or slightly reduced compared to the uninstructed case.

We could not identify *a priori* patients with significant interfraction diaphragm variability. All the patients were able to tolerate the longer simulation and approximately 5 min longer treatment time and were willing and able to follow voice prompting. Patient 4_Li developed ascites during treatment, a medical reason for a superior diaphragm shift, and the stomach contents of 2_Li may have differed between simulation and treatments. Otherwise, we saw no distinction between patients with large and small interfraction changes. Large systematic or random changes in *D* were of particular concern for the liver patients because they were selected for gating to reduce PTV margins. As a result of the diaphragm films, the PTVs and field sizes were increased for 5 liver cancer patients and 1 lung cancer patient.

In the future, respiration‐correlated CT (RCCT) imaging^(22^ ^–^ ^25,^, ^27,^, ^28)^ of the GTV and diaphragm will be helpful. RCCT acquires CT images for breathing phases covering the whole breathing cycle and shows GTV displacement and deformation and the GTV's relation to the diaphragm. If contrast is used to visualize liver tumors, precise timing of the RCCT study relative to the contrast administration is crucial. This may be possible with the multislice scanner/cine method described by Pan et al.,^(24)^ which acquires 1 cm of data (four 2.5‐mm slices) per breathing cycle (~5 s). While end‐exhale images could be used for planning, the envelope of the GTVs at all breathing phases within the gate would determine an intra‐fractional margin for motion within the gate. The population distribution of diaphragm displacements acquired from the diaphragm films of previous patients together with the relationship between diaphragm and GTV from the patient's RCCT scan would determine a patient‐specific initial margin for interfractional variability. The PTV would include further margin for setup error. After approximately five daily diaphragm films (one week), the interfraction margin could be changed on the basis of the observed diaphragm displacements. For a nondeforming GTV and a large systematic diaphragm shift with small random variation, an isocenter shift might be sufficient. For patients with small interfraction variability, margin reduction might be possible. Surveillance of interfraction variability would continue at reduced frequency, determined in part by imaging dose, to identify large diaphragm variability later in the course of treatment. Currently, we cut back to 1 or 2 per week. If we could conveniently use the EPID, the imaging dose could be reduced to 1 MU per diaphragm “film.” Anticipating this capability, we are investigating the applicability of alternative monitoring and correction strategies.^(29)^

RCCT will not solve all interfractional variation problems. If $D_{\text{pt}}$ is outside the range of diaphragm displacements in the RCCT session, as for the 7 patients whose diaphragms at treatment were significantly superior to their end‐exhale position at simulation, the GTV displacement would have to be extrapolated from the RCCT images. For some patients, the relationship between diaphragm and GTV displacements may change over the course of treatment. And in general, it would be preferable to image the tumor rather than the diaphragm surrogate. Developing the ability to do this conveniently, at weekly or more frequent intervals over the course of treatment, is a subject of active research.^(4,30–39)^

## V. CONCLUSION

Interfractional reproducibility of internal thoracic anatomy, as indicated by the diaphragm, is not assured by respiratory gating based on the motion of an external marker. Radiographic surveillance of gating patients throughout their course of treatment is needed to monitor interfractional variability.

## ACKNOWLEDGMENTS

Supported in part by grant #PO1–CA–59017 from the National Cancer Institute, National Institutes of Health. We also acknowledge a research agreement with Varian Medical Systems.
